# The attitude towards the forest and satisfaction with life of Polish students

**DOI:** 10.1371/journal.pone.0302247

**Published:** 2024-04-16

**Authors:** Anna Koprowicz, Robert Korzeniewicz, Wojciech Pusz, Marlena Baranowska

**Affiliations:** 1 Department of Pedagogy, Pomeranian University in Słupsk, Słupsk, Poland; 2 Department of Silviculture, Poznan University of Life Sciences, Poznań, Poland; 3 Department of Plant Protection, Wrocław University of Environmental and Life Sciences, Wrocław, Poland; University of KwaZulu-Natal College of Health Sciences, SOUTH AFRICA

## Abstract

The aim of the study is to determine the connection between the attitude towards the forest and life satisfaction of students. The study was conducted on a sample of 650 students from Polish universities using The Satisfaction With Life Scale and an original questionnaire measuring the approach towards the forest–LAS scale. There are 3 subscales which measured: the perceived benefits from spending time in the forest; the degree of involvement in exploring the forest and working to its advantage; fears connected with the forest. The scale indicated good psychometric properties. Its reliability expressed by Cronbach’s alpha coefficient is at the level of 0.90 (0.93 for Benefits scale; 0.87 for Involvement and 0.79 for Fears). The results of the statistical analysis have led to conclusions confirming the relationship between life satisfaction and the results of LAS scale. People satisfied with their lives are more involved in exploring the forest, they enjoy more benefits connected with forest recreation and express fewer fears. Forestry students are characterised by the highest involvement and the lowest level of fears connected with the forest. As far as life satisfaction is concerned, they are in the lead among Polish students just behind students of medical and artistic studies.

## 1. Introduction

The development of positive psychology sparked an increased interest of scholars to seek the determinants of human happiness. The issue of what causes life satisfaction despite hard living conditions has caused more and more ponderings. In the 1990s, the studies concerning subjective well-being (SWB) started to appear. It is considered that SWB is a construct composed of cognitive as well as emotional components [1]. SWB includes three factors: positive affect, negative affect and life satisfaction. Life satisfaction (LS) is distinguished among the factors which refers to the cognitive assessment of one’s life as a whole [2]. Life satisfaction is defined as “a global assessment of a person’s quality of life according to his chosen criteria” [Shin, Johnson, 1978, p. 478, after: [3]. The definition emphasises the cognitive and subjective nature of life satisfaction. The subjective assessment of life situation, in comparison to the life situation of others, is conducted through the prism of values significant for an individual together with their resources and limitations and is thought to be the measure of quality of life [4].

It is indicated that life satisfaction depends on numerous factors as well as complex cultural, personal and environmental relationships [1]. Apart from the factors connected with objective conditions in which the individual lives or the life events, life satisfaction is also connected with psychological variables. Studies have proven the connection between LS with pro-health behaviours [4], temperament [5], positive affect, self-esteem [4], optimism, extraversion [2]. Moreover, the negative relationship between LS and negative affect, pessimism, depression, perceived stress, suicide ideation, neuroticism has also been confirmed (ibidem).

The studies conducted within the domain of environmental psychology have confirmed the link between well-being and life satisfaction with the contact with nature [6] The role of nature for human health and well-being is an undeniable fact [7–11].

A broad analysis of benefits which people derive from the contact with nature has been presented by M. Kuo [12] among others, who described 21 pathways from nature to health, including environmental factors, physiological and psychological states, as well as behaviours or conditions. Apart from describing the obvious connection between the level of afforestation and the quality of air, the author indicates such outcomes of contact with nature as decreasing the symptoms of anxiety and depression, stress reduction, improving concentration and vitality, boosting immune system against infections, lowering the risks connected with cardiovascular diseases and cancers, increased physical activity which lowers the risks of obesity. Moreover, nature helps strengthen social ties and even lowers the level of violence. In addition, trees in urban areas contribute to the improvement of the inhabitants’ quality of life [13, 14]. It has been proven by the MillionTreesNYC project. B.A. Jones [15] studied the impact of the program on the subjective well-being of inhabitants. His results indicate that life satisfaction improves as the number of planted trees increases; however, in the period between December and February when the trees are without the foliage this impact is lowered. Spending time in nature can be a transcendental experience connected with exposure to the elements, a force greater than an individual person which, in turn, is a nudge towards prosocial behaviours [16] Unique research conducted on a group of over 20000 participants proves that people are happier in all green and natural habitat types than in urban spaces [17]. In turn, city residents feel more satisfied with life if they have a garden or urban green areas nearby [18, 19]. According to the biophilia theory, love of nature allows people to overcome feelings of isolation and loneliness, and promotes a sense of connection and belonging to the natural world and empathy with other living beings [20] Connectedness to nature positively correlates with life satisfaction [21] and promotes lower stress level [22] Moreover, this variable is associated with a sense of life satisfaction at a similar level as marriage, education or income level [23].

The previously conducted studies indicate the benefits of spending time in the forest for physical and mental well-being and health in general. It has been shown that the level of happiness increases while spending time in nature and also noticed an increase in experienced happiness when people spend time in the forest. In addition, it has been noticed that even passive contact with natural environment contributes to the formation of positive emotions [24] and lowers stress levels [25]. People who spend more time in the nature during the pandemic indicated fewer symptoms of depressive disorders than people less exposed to nature [26]. Moreover, it has been emphasised that people with high emotional sensitivity can derive considerable benefits from contact with nature which, in their case, possesses special regenerative properties [25]. During the pandemic, special attention was paid to the importance of contact with nature, in particular forest recreation, to reduce the long-term consequences of covid-19 [27].

A general improvement of health, social relations or environmental conditions correlate definitely to the subjective assessment of life satisfaction.

For many people contact with nature becomes a passion which encourages them to choose profession connected with nature. Despite so many studies on the beneficial effects of nature on human health and well-being, it is still difficult to find studies that combine attitudes towards the forest and life satisfaction with the chosen field of study.

The presented research aimed to find out the relationship between the attitude towards the forest and satisfaction with life of Polish students, taking into account the field they are studying.

## 2. Materials and methods

### 2.1. Aim, problems and hypotheses

The aim of the study was to determine the relationship between the attitude to the forest and life satisfaction of Polish students. In particular, it was pondered whether students of forestry studies, which a priori assume a positive attitude to the forest, are more satisfied with their lives than students of other studies.

Research questions have been formulated as follows:

Is there a relationship between the attitude to the forest of Polish students and their life satisfaction?

Does the choice of studies differentiate the respondents in terms of the attitude to the forest and their life satisfaction?

To such formulated research questions, the following hypotheses have been constructed:

H1. The students’ more positive attitude to the forest, the greater their life satisfaction is.

This hypothesis is supported by biophilia theory, which emphasizes that the feeling of unity with nature makes people happier [20]. In addition, research conducted by Biedenweg and others [28] shows that involvement in learning about nature (i.e., one of the items in the scale we developed to measure attitudes toward the forest) is related to life satisfaction.

H2. Forestry students are characterized by a more positive attitude to the forest than students of other studies. These students also better assess their life satisfaction than other respondents. The first part of this hypothesis seems obvious—it would be difficult to assume that a person with a negative attitude toward the forest would decide to choose forestry as a field of study and future profession. So far, however, this assumption has not been empirically verified, especially with regard to the relationship of attitudes toward the forest presented by forestry students with their sense of satisfaction with life.

### 2.2. Participants

Six hundred and fifty respondents participated in the study, they were students of different studies, 428 of which were women. The age of the respondents was between 19 and 24 (mean: 21,71). The students of forestry studies and medical studies constituted the most numerous groups. Other respondents were students of social and humanistic studies, life sciences studies, technical and science studies and artistic studies. Table 1 shows the sociodemographic characteristic of the sample.

**Table 1:** Sociodemographic characteristic of the sample.

		N	%
Gender	Female	428	65,85
	Male	222	34,15
Age	19–20	146	22,46
	21–22	223	34,31
	23–24	281	43,23
Place of residence	Village	311	47,85
	Small town (up to 20 thousand residents)	102	15,69
	Medium city (between 20 and 100 thousand residents)	90	13,84
	Large city (over 100 thousand residents)	147	22,62
Field of studying	Social and humanistic	84	12,92
	Forestry	267	41,08
	Art	39	6
	Medical	158	24,31
	Life sciences	55	8,46
	Technical	47	7,23

### 2.3. Measures

The study was conducted using diagnostic survey method. The adopted tools were:

1. Socio-demographic survey which enabled collecting the basic data on the structure of surveyed group.
2. The Satisfaction with Life Scale (SWLS) by E. Diener, R.A. Emmons, R.J. Larsen and S. Griffin which was adapted to Polish realities by Z. Juczyński [29]. The tool consists of 5 statements to which the respondents have to refer to using the 7-point scale; from 1 –strongly disagree to, 7 –strongly agree. The scale is considered to be characterised with satisfactory psychometric properties–the reliability of the scale is measured by Cronbach’s alfa coefficient which is 0.81; the theoretical validity was established by correlating the results of other tests connected with life satisfaction in general and on the basis of factor analysis, which confirmed the occurrence of a single factor which explains 56.6% of variance.
3. LAS scale (“Las” means “forest” in Polish)—independently prepared tool for measuring the attitude towards the forest. The first stage of creating the tool was to formulate a set of 70 statements by 2 psychologists and 2 foresters, and they believe the statements can be useful in studying the attitude to the forest. A coherence construction was maintained which assumed the possibility of taking a stance to each of the statements following Likert scale, where 1 means “strongly disagree” and 5 “strongly agree”. In the second step 5 competent assessors, psychologists and academic teachers, were asked to express their opinion whether the statements are really useful in studying the approach towards the forest. The next step encompassed choosing 52 statements (items) to which the assessors agreed to their usefulness in at least 80%. Such a prepared questionnaire was used to study 518 people. The questionnaire was shared on the Internet using Google Forms between September and December 2020. The answers collected in such a way were subjected to a factor analysis. After adopting scree test and Kaiser criterion 3 independent factors of approach to the forest were identified. Only those items, whose factors loadings were greater than 0.50, were qualified to the final version of the questionnaire. In the first factor, 8 statements concerning the experience of pleasure and health benefits connected with spending time in the forest, such as: “I rest and relax in the forest very well”, “The forest helps me to improve my health”, “I feel happy in the forest”. This factor has been named as “Benefits”. The second factor included 8 statements, such as: “I take part in tree planting campaigns”, “I am aware of the problems connected with forest management”, or “I am interested in nature–I am keen on learning about various tree species or reading about animals”. This factor has been named “Involvement”. The third factor, named “Fears”, constituted of 4 statements, for example: “I am afraid to go to the forest because of ticks” or “I worry that I will get lost in the forest”. The tool constructed in such a way, consisting of 20 statements, is characterised by good reliability expressed by Cronbach’s alpha coefficient at the level of 0.90 (0.93 for Benefits scale; 0.87 for Involvement and 0.79 for Fears) (S1 Appendix).

### 2.4. Procedure

The survey was conducted in the spring and summer of 2021. Restrictions put in place at the time to prevent the spread of the COVID-19 pandemic prevented direct contact with students, so the study was conducted via the Internet. The survey questionnaire was made available as a link to the Google Forms website. The link was sharing means of social media (Facebook), and academic teachers who work with students of the particular studies. Five universities with medical, arts, forestry, life sciences, technical sciences and social sciences majors were randomly selected from among universities in Poland. Information about the survey, along with a request to share a link to the surveys with students, was sent to deans of faculties and heads of institutes. All the participants were familiarised with the aim of the study and consciously agreed to take part in it. Informed written consent was obtained from each participant. Before participants started completing the online research survey, they had to read the information about the project and its aims and select the "I agree to participate in the study" option. The data were analyzed anonymously. The study was designed in accordance with guidelines for ethical conduct of research. All participants were of legal age and were able to give informed consent to participate in the study. They were also assured that they could withdraw from the study without any negative consequences. The study did not obtain any personal data or email addresses enabling the identification of respondents. None of the examined persons was exposed to harmful and traumatizing factors. The research procedure was approved by the University Committee for Research Ethics in Pomeranian University in Słupsk (opinion no. UKEBN/4/2023).

### 2.5. Analyses

Statistical calculations were conducted using Statistica 10 software. In the process of constructing the tool (LAS), factor analysis was used, taking into account the scree plot and Kaiser criterion. Additionally, the Cronbach’s coefficient was used to calculate the reliability of the LAS. The Kolmogorov-Smirnov test was used to check the normality of the distribution of variables’ values. To examine the correlation between life satisfaction and attitudes towards the forest t-test was used. As far as the SWLS results are concerned, whose distribution is similar to normal and the condition of homogeneity of variance was preserved, the analysis of variance (ANOVA) was used for calculations. In the case of achieved results a nonparametric test (Kruskal-Wallis one-way analysis of variance by ranks) was adopted for particular factors of LAS scale.

## 3. Results

The correlation analysis, between the achieved results in the particular factors of the LAS scale and the overall SWLS results, indicates a slight, but statistically significant, connection. The benefits that the participants perceive while spending time in the forest correlate with a general feeling of life satisfaction (r = 0.12; p = 0.006). Moreover, the greater involvement of the respondents in exploring the forest and working to its benefit, the greater feeling of life satisfaction (r = 0.16; p = 0.001). Life satisfaction correlates negatively with the fears which the respondents experience towards the forest; the greater feeling of life satisfaction, the lower the fears (r = -0.14; p = 0.001). The results confirm the hypothesis which linked the attitude to the forest and the feeling of life satisfaction.

**Fig 1:**
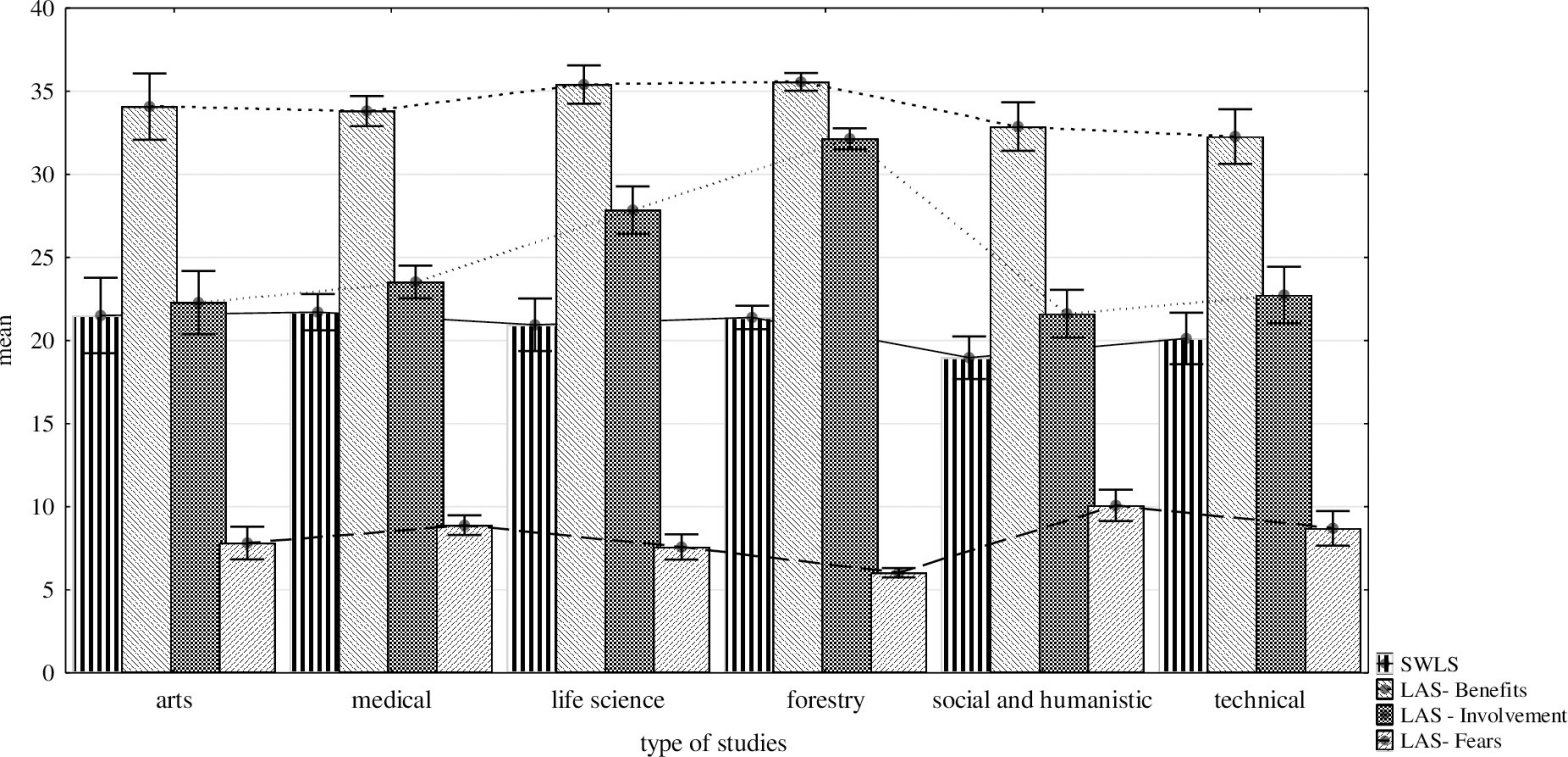
Mean results achieved in LAS scale and SWLS including the type of studies.

The next stage of the analysis attempted to establish whether the type chosen studies differentiates the respondents in reference to the analysed variables. The results achieved by the students of particular studies have been presented in Fig 1.

The comparison of means (Table 2) reveals that students of medical studies are most satisfied with their lives, and next are the students of artistic and forest studies. The lowest on the scale are the students of humanistic and social studies–their life satisfaction is below the average.

**Table 2:** Type of studies and life satisfaction–the results of analysis of variance.

Type of studies	Satisfaction with life			df	F	p-value
	N	mean	SD			
Social and humanistic	84	18.96	5.89	644	2.689	0.002
Forestry	267	21.39	5.87			
Art	39	21.51	6.99			
Medical	158	21.71	6.95			
Life sciences	55	20.95	5.84			
Technical	47	20.12	5.29			
General	650	21.03	622			

The post-hoc analysis with the least significant difference test (see S2 Appendix) indicate that statistically significant differences occur between humanists and artists, future medics and foresters. The students of social and humanistic studies are definitely less satisfied with their lives than the students of medicine, forestry or artistic studies. Among the students of other studies, the differences did not reach the level of statistical significance.

The statistically significant difference occurred also in reference of respondents’ attitude to the forest depending on their types of studies (Table 3). The type of studies differentiates the respondents in all three factors which are included in LAS scale. Forestry students acknowledge more benefits of spending time in the forest, and at the same time express fewer fears connected with it. In comparison with other respondents, they are more involved which partially relates to the specificity of the studied subject as well as personal interests which motivated the respondents to choose this profession.

**Table 3:** The type of studies and the attitude to the forest—The results of Kruskal-Wallis test.

**Type of studies**	**LAS—benefits**					
	**Mean**	**SD**	**Sum of ranks**	**Average rank**	**H**	**p-value**
Social and humanistic	32,880	6,729	23983.0	285.512	22.908	0.004
Forestry	35,57	4,375	95624.5	358.144		
Art	34,077	6,149	12584.5	322.679		
Medical	33,816	5,746	48763.0	308.627		
Life sciences	35,400	4,271	19126.0	347.746		
Technical	32,276	5,582	11494.0	244.553		
	**LAS—involvement**					
	**Mean**	**SD**	**Sum of ranks**	**Average rank**	**H**	**p-value**
Social and humanistic	21,619	6,647	16162.5	192.411	252.851	0.001
Forestry	32,138	5,264	122497.5	458.792		
Art	22,282	5,884	12584.5	322.679		
Medical	23,525	6,270	36558.5	231.383		
Life sciences	27,854	5,293	18749.0	340.891		
Technical	22,745	5,799	9843.5	209.436		
	**LAS—fears**					
	**Mean**	**SD**	**Sum of ranks**	**Average rank**	**H**	**p-value**
Social and humanistic	10,083	4,358	36409.5	433.446	115.775	0.001
Forestry	6,030	2,304	62946.5	235.755		
Art	7,820	3,025	13693.5	351.115		
Medical	8,892	3,726	61798.5	391.129		
Life sciences	7,581	2,832	18580.0	337.818		
Technical	8,702	3,532	18147.0	386.106		

The post-hoc analysis revealed that as far as the benefits and fears connected with the forest are concerned the foresters-to-be differ statistically significantly from the students of social and humanistic, medical, technical and science studies. By comparison with the students of life science and artistic studies the differences are not statistically significant. As far as involvement is concerned, the satisfactory level of statistical significance was the difference between the students of forestry students and all the other respondents.

## 4. Discussion

In the light of the presented study the existence of the link between attitudes to the forest and life satisfaction has been confirmed. The more benefits students experience from forest recreation and the more they involve in exploring the forest and working to its benefit, the higher the level of life satisfaction. In contrast, anxiety related to spending time in the forest is negatively related to life satisfaction. We also found that students in different majors differed in both their attitudes toward the forest and their perceived satisfaction with life. Our study is a part of a trend of research confirming the relationship between being in nature and perceived life satisfaction. Its new element, little present in previous empirical studies, was to pay attention to the forest. The forest environment took on particular importance during the COVID-19 pandemic—the forest was one of the few spaces where tired, stressed-out people could spend time without risking infection from the disease. Beyond that, we developed a tool that made it possible to explore three important aspects of attitudes toward the forest: benefits, involvement, and fears.

The studies presented here have revealed that the fears connected with the forest correlate negatively with LS which means that the more fears are felt by respondents, the lower is their live satisfaction. The fears of spending time in the forest, such as fear of ticks and wild animals or the fear of getting lost may be related to personality traits. Numerous studies focused on the links between personality (Big Five Factors) and LS have confirmed that the higher level of neurotics among the respondents is, the lower life satisfaction [30]. However, in some situations the forest ecosystems can constitute a real danger for human health [31] Avoiding the forest can attest to the awareness and responsibility of the respondents, especially that many organisms which live in the forest can cause allergies and unpleasant skin reactions [32–35].

The second of the hypothesis stated in the study assumed that forestry students are characterised by a more positive attitude to the forest and, at the same time, greater life satisfaction than the students of other courses. The hypothesis has been partially confirmed. Foresters-to-be are significantly more involved in exploring the forest and working to its benefit than future doctors, computer experts or psychologists. K. Biedenweg [28] among others, draws attention to the role of being involved in natural environment and its influence on life satisfaction. Scholars [28]) have indicated that life satisfaction relates to such factors as involvement in natural environment or psychological benefits of spending leisure time in fresh air, outdoors recreation, or social and cultural events connected with the environment. In the scope of felt benefits of spending time in the forest or the fears connected with it, foresters differ from the students of humanistic, social studies, medical or science and technical studies, they are, however, similar to their peers who study life sciences and artistic studies.

Furthermore, it occurred that the students of forestry have a high level of life satisfaction. The only better results in SWLS were achieved by the students of medical and artistic studies, which can be connected with the specificity of Polish higher education system. Such professions as a doctor or a dentist are perceived as prestigious and profitable which is the reason why medical studies are so popular among secondary school graduates. This also explains such a strong competition during the recruitment process. Getting admitted to medical school is prestigious enough, studying and passing the difficult exams also improves self-esteem which then translates to life satisfaction [36]. Artistic studies may not be so prestigious and are not connected with the prospects of high income in the future; however, getting admitted to art school requires a special artistic talent which will be appreciated and assessed by specialists. This also seems to be a factor which contributes to raising self-esteem of the candidates and students. Life satisfaction is connected with work satisfaction [37], hence the dissatisfaction with the studies and student internships can lead to disappointment with the chosen profession and at the same time lower general life satisfaction. The observations should become a challenge for academic teachers who can undeniably influence the attractiveness of the classes.

A disturbing phenomenon which was revealed during the studies is a low level of life satisfaction among the students of social and humanistic studies. Among this group there are future psychologists, educators, social workers–people whose work is based on helping others. It can be assumed that people who choose such professions are usually more sensitive, they experience suffering more intensively hence they can be more empathic towards the suffering of others. Furthermore, it can be speculated that during lockdowns those people with a strong need for affiliation (which includes social sciences students) could particularly feel the effects of social isolation which impacts their life satisfaction. The thesis seems worth exploring and subjecting it to empirical verification. However, it is difficult to avoid the thoughts that people with a low level of life satisfaction are more susceptible to depressive disorders and, thus, their ability to offer help and psychological support is lowered. Understanding the correlation between life satisfaction of students of social studies with their features of character, ways of dealing with stress and frustration, or even with the symptoms connected with disorders will allow further exploration of the specificity of their functioning and contribute to creating a support system for universities.

It seems interesting to continue in-depth study of the correlations between attitude to the forest and mental issues such as mood or anxiety disorders. The tool which was prepared for this study (LAS scale) which is characterised by good psychometric properties can be adopted and used for further academic studies.

The existence of the correlation between attitude to the forest and life satisfaction can be also used practically. First of all, it is necessary to ensure environmental education of children: making them aware of the benefits of forest recreation, as well as providing reliable information about the possible dangers (it will contribute to lowering the neurotic fear of spending time in the forest); including children in working for the benefit of the forest (it will increase the involvement into exploring and learning about the forest); arouse children’s interest in nature and present forest recreation as an attractive alternative to other activities. Forest schools can play an important role here. Harris’ research [38] shows that staying in such schools lowers the level of anxiety and promotes the development of pro-ecological behaviour and the ethos of caring for forest. Such actions, if they bring the expected outcome by shaping a positive attitude to the forest, can prove to be a valuable investment in life satisfaction of future generations.

### 4.1. Limitations

One of the limitations of the presented studies is the method of collecting the data via the Internet. Among numerous advantages, such as the possibility of reaching out to a considerable number of research sample from various and distant places, the method is also connected with certain risks connected with the inability to verify the data provided by the respondents in the questionnaire. Another limitation connected with the results was the disproportion in the number of respondents from each group. The discrepancies in the number of students from forestry and, for example artists, is the reason why the findings should be treated with a degree of caution.

Although the universities invited to participate in the study were randomly selected, we could not control how the university teachers selected the students, and therefore the entire selection of the research sample cannot be considered random. Therefore, the results obtained are not representative of the general population of Polish students. Moreover, the study focused on Polish students, so it should not be generalized to other groups. When comparing the results of this study with similar ones conducted in other countries, one should take into account, among others, the situation of young people studying in Poland and the specificity of the Eastern European region.

## 5. Conclusions

The forest plays various roles and functions in human life. Not only are the roles connected with exploiting the forest resources, but also with social and health functions. The results of the studies reveal the relationship between attitudes to the forest and life satisfaction. People who derive benefits from the forest, are involved in exploring nature and feel fewer fears connected with forest recreation are, at the same time, more satisfied with their lives. It proves that the forest and spending time there influence the well-being temporarily (higher oxygen levels, enhanced mood, calms and relaxes), but they also have long-term ramifications by bringing people closer to satisfaction with life. The research presented in this article confirms that people who like the forest and engage in learning about it enough to choose an educational path that will help them become professionally involved with the forest in the future declare greater satisfaction with their lives than young people who choose social or technical studies.

Despite the limitations described above, the study may contribute to a better understanding of the relationship between attitude towards the forest and life satisfaction. It may also lead to the conclusion that investing in nature education, which promotes better recognition of the benefits of staying in nature, may contribute to greater involvement in learning about the forest and reducing the fear associated with being there, which will result in an improvement in the quality of life of Poles.

## Supporting information

S1 Appendix The LAS scale. (DOCX)

S2 Appendix Supplementary data—Post-hoc analysis. (DOCX)
