# Supplementary material for: The attitude towards the forest and satisfaction with life of Polish students
Source: PLoS One. 2024 Apr 16;19(4):e0302247. doi: 10.1371/journal.pone.0302247 (PMC11020966; doi:10.1371/journal.pone.0302247)
Supplement: S1 Appendix — (DOCX) [file pone.0302247.s001.docx]

**S1 Appendix**

LAS scale

**LAS**

We are interested in your attitude to the forest. Please take a stand on the following statements by marking the appropriate answer. There are no good or bad answers. All answers are correct as long as they reflect your true feelings. The questionnaire is completely anonymous and the results will be used for academic analyses.

|  |  | Strongly agree | Agree | Neither agree nor disagree | Disagree | Strongly disagree |
| --- | --- | --- | --- | --- | --- | --- |
| 1. | I like spending time in the forest | 5 | 4 | 3 | 2 | 1 |
| 2. | I think I would do a good job as a forester | 5 | 4 | 3 | 2 | 1 |
| 3. | I relax and rest very well in the forest | 5 | 4 | 3 | 2 | 1 |
| 4. | I’m interested in nature – I like learning about different tree species, read about animals, watch wild life documentaries etc. | 5 | 4 | 3 | 2 | 1 |
| 5. | I’m afraid to go to the forest because of the ticks | 5 | 4 | 3 | 2 | 1 |
| 6. | During the pandemic a walk in the forest helps me relax and distance myself from fears | 5 | 4 | 3 | 2 | 1 |
| 7. | I’m aware of the problems connected with forest economy | 5 | 4 | 3 | 2 | 1 |
| 8. | I feel happy in the forest | 5 | 4 | 3 | 2 | 1 |
| 9. | I eagerly take part in various campaigns organised by foresters, such as feeding animals or cleaning up the forest | 5 | 4 | 3 | 2 | 1 |
| 10. | The sounds of forest animals evoke fear in me | 5 | 4 | 3 | 2 | 1 |
| 11. | I free myself from thinking about my problems in the forest | 5 | 4 | 3 | 2 | 1 |
| 12. | I like talking to people who work in the forest | 5 | 4 | 3 | 2 | 1 |
| 13. | I feel more relaxed and calmer after walking in the forest | 5 | 4 | 3 | 2 | 1 |
| 14. | I visit places which are particularly interesting such as nature reserves | 5 | 4 | 3 | 2 | 1 |
| 15. | I worry that I will get lost in the forest | 5 | 4 | 3 | 2 | 1 |
| 16. | Walking in the forest relaxes me | 5 | 4 | 3 | 2 | 1 |
| 17. | I like forest educational paths | 5 | 4 | 3 | 2 | 1 |
| 18. | The forest contributes to the improvement of my health | 5 | 4 | 3 | 2 | 1 |
| 19. | I take part in the campaigns of planting the forest | 5 | 4 | 3 | 2 | 1 |
| 20. | I’m afraid to go to forest because of wild animals | 5 | 4 | 3 | 2 | 1 |

**Benefits**: 1; 3; 6; 8; 11; 13; 16; 18

**Involvement**: 2; 4; 7; 9; 12; 14; 17; 19

**Fears**: 5; 10; 15; 20 (when calculating the total scores for the attitude to the forest, the answers in Fears subscale should be recoded according to the formula: answer strongly agree=1 point, agree=2, neither agree nor disagree=3, disagree=4, strongly disagree=5)
