# Supplementary material for: The attitude towards the forest and satisfaction with life of Polish students
Source: PLoS One. 2024 Apr 16;19(4):e0302247. doi: 10.1371/journal.pone.0302247 (PMC11020966; doi:10.1371/journal.pone.0302247)
Supplement: S2 Appendix — (DOCX) [file pone.0302247.s002.docx]

**S2 Appendix**

Supplementary data - post-hoc analysis

| **Satisfaction with Life Scale** | | | | | | |
| --- | --- | --- | --- | --- | --- | --- |
| **Type of studies** | **1.**  M=18.964 | **2.**  M=21.513 | **3.**  M=21.390 | **4.**  M=20.945 | **5.**  M=20.128 | **6.**  M=21.709 |
| **1.**  **social and humanistic** |  | **0.0337⃰** | **0.0018⃰** | 0.0650 | 0.3018 | **0.0011⃰** |
| **2.**  **artistic** | **0.03****37 ⃰** |  | 0.9074 | 0.6611 | 0.3011 | 0.8592 |
| **3.**  **forestry** | **0.0018⃰** | 0.9074 |  | 0.6277 | 0.1972 | 0.6068 |
| **4.**  **life sciences** | 0.0650 | 0.6611 | 0.6277 |  | 0.5055 | 0.4304 |
| **5.**  **technical** | 0.3018 | 0.3011 | 0.1972 | 0.5055 |  | 0.1240 |
| **6.**  **medical** | **0.0011⃰** | 0.8592 | 0.6068 | 0.4304 | 0.1240 |  |
| **LAS scale - Benefits** | | | | | | |
| **Type of studies** | **1.**  M=32.881 | **2.**  M=34.077 | **3.**  M=35.566 | **4.**  M=35.400 | **5.**  M=32.277 | **6.**  M=22.816 |
| **1.**  **social and humanistic** |  | 0.2420 | **0.0001⃰** | **0.0060⃰** | 0.5292 | 0.1891 |
| **2.**  **artistic** | 0.2420 |  | 0.0999 | 0.2309 | 0.1153 | 0.7823 |
| **3.**  **forestry** | **0.0001⃰** | 0.0999 |  | 0.8320 | **0.0001⃰** | **0.0009⃰** |
| **4.**  **life sciences** | **0.0060⃰** | 0.2309 | 0.8320 |  | **0.0029⃰** | 0.0554 |
| **5.**  **technical** | 0.5292 | 0.1153 | **0.0001⃰** | **0.0029⃰** |  | 0.0791 |
| **6.**  **medical** | 0.1891 | 0.7823 | **0.0009⃰** | 0.0554 | 0.0791 |  |
| **LAS scale - Involvement** | | | | | | |
| **Type of studies** | **1.**  M=21.619 | **2.**  M=22.282 | **3.**  M=32.139 | **4.**  M=27.855 | **5.**  M=22.745 | **6.**  M=23.525 |
| **1.**  **social and humanistic** |  | 0.5547 | **0.0001⃰** | **0.0001⃰** | 0.2862 | **0.0150⃰** |
| **2.**  **artistic** | 0.5547 |  | **0.0001⃰** | **0.0001⃰** | 0.7123 | 0.2302 |
| **3.**  **forestry** | **0.0001⃰** | **0.0001⃰** |  | **0.0001⃰** | **0.0001⃰** | **0.0001⃰** |
| **4.**  **life sciences** | **0.0001⃰** | **0.0001⃰** | **0.0001⃰** |  | **0.0001⃰** | **0.0001⃰** |
| **5.**  **technical** | 0.2862 | 0.7123 | **0.0001⃰** | **0.0001⃰** |  | 0.4174 |
| **6.**  **medical** | **0.0150⃰** | 0.2302 | **0.0001⃰** | **0.0001⃰** | 0.4174 |  |
| **LAS scale - Fears** | | | | | | |
| **Type of studies** | **1.**  M=10.083 | **2.**  M=7.820 | **3.**  M=6.030 | **4.**  M=7.582 | **5.**  M=8.702 | **6.**  M=8.892 |
| **1.**  **social and humanistic** |  | **0.0003⃰** | **0.0001⃰** | **0.0001⃰** | **0.0174⃰** | **0.0057⃰** |
| **2.**  **artistic** | **0.0003⃰** |  | **0.0011⃰** | 0.7202 | 0.2014 | 0.0601 |
| **3.**  **forestry** | **0.0001⃰** | **0.0011⃰** |  | **0.0011⃰** | **0.0001⃰** | **0.0001⃰** |
| **4.**  **life sciences** | **0.0001⃰** | 0.7202 | **0.0011⃰** |  | 0.0768 | **0.0087⃰** |
| **5.**  **technical** | **0.0174⃰** | 0.2014 | **0.0001⃰** | 0.0768 |  | 0.7191 |
| **6.**  **medical** | **0.0057⃰** | 0.0601 | **0.0001⃰** | **0.0087⃰** | 0.7191 |  |

⃰ p ≤ 0,05
